# Flower Bulb Waste Material is a Natural Niche for the Sexual Cycle in *Aspergillus fumigatus*

**DOI:** 10.3389/fcimb.2021.785157

**Published:** 2022-01-21

**Authors:** Jianhua Zhang, Paul E. Verweij, Antonius J. M. M. Rijs, Alfons J. M. Debets, Eveline Snelders

**Affiliations:** ^1^ Laboratory of Genetics, Wageningen University & Research, Wageningen, Netherlands; ^2^ Radboud University Medical Center (Radboudumc), Nijmegen, Netherlands; ^3^ Canisius-Wilhelmina Ziekenhuis (CWZ) Center of Expertise for Mycology, Radboud University Medical Center (Radboudumc), Nijmegen, Netherlands

**Keywords:** *Aspergillus fumigatus*, *Neosatorya fumigata*, sexual cycle, cleistothecia, ascospores

## Abstract

With population genetic evidence of recombination ongoing in the natural *Aspergillus fumigatus* population and a sexual cycle demonstrated in the laboratory the question remained what the natural niche for *A. fumigatus* sex is. Composting plant-waste material is a known substrate of *A. fumigatus* to thrive and withstand temperatures even up to 70°C. Previous studies have shown indirect evidence for sexual reproduction in these heaps but never directly demonstrated the sexual structures due to technical limitations. Here, we show that flower bulb waste material from stockpiles undergoing composting can provide the conditions for sexual reproduction. Direct detection of ascospore structures was shown in agricultural flower bulb waste material by using a grid-based detection assay. Furthermore, we demonstrate that ascospores can germinate after exposure to 70°C for up to several days in contrast to asexual conidia that are unable to survive a two-hour heat shock. This indicates a sufficient time frame for ascospores to survive and escape composting stockpiles. Finally, sexual crosses with cleistothecium and viable ascospore formation could successfully be performed on flower bulb waste material. Recombination of *A. fumigatus* can now be explained by active sexual reproduction in nature as we show in this study that flower bulb waste material provides an environmental niche for sex.

## Introduction

*Aspergillus fumigatus* is known as a fungus of concern for human health particularly in immunocompromised patients. This fungus has a ubiquitous presence in our environment and healthy individuals exhibit high innate immunity to prevent *Aspergillus* diseases due to efficient clearing of the spores from the lungs (Hohl and Feldmesser, 2007). The most lethal manifestation of *Aspergillus* diseases includes invasive aspergillosis (IA), an infection that is characterized by tissue invasive growth of the fungus. IA is known to develop in patients with neutropenia, but in recent years cases are increasingly observed in nonneutropenic critically ill patients including those with severe influenza and coronavirus disease 2019 (COVID-19) (Schwartz et al., 2020; Chong and Neu, 2021). A major improvement in the management of *Aspergillus* diseases was made with the introduction of the triazole class, including itraconazole, voriconazole, posaconazole and isavuconazole. These triazoles have become evidence-based first choice treatment options for both treatment and prophylaxis of *Aspergillus* diseases. Management of IA has however remained difficult despite the availability of the triazoles. Diagnosis is often challenging because clinical symptoms and biomarkers are not always specific for IA, patient groups have become more heterogenous, and because of the emergence of acquired triazole resistance. Although triazole resistance was long neglected to be an uncommon phenomenon (Patterson and Strek, 2014), over the past decades triazole resistance has increased substantially in some geographic regions, mainly due to *A. fumigatus* isolates harboring TR_34_/L98H and TR_46_/Y121F/T298A mutations in the triazole target gene; the *cyp*51A gene. Triazole resistance was shown to be associated with treatment failure and 20% excess mortality in patients with IA (Lestrade et al., 2019). The most likely explanation for the spread of these tandem repeat(TR)-mediated resistance mechanisms in *A. fumigatus* is the use of triazole fungicides in agriculture (Verweij et al., 2009; Schoustra et al., 2019a). As the number of resistance mutations and variations increases over time, understanding the relationship between triazole fungicide exposure and the development of triazole resistance mutations is critical to contain this problem.

In order to optimally create genetic variation for adaptation to a triazole-containing environment, *A. fumigatus* needs to complete its reproduction cycle. *A. fumigatus* may benefit from three reproduction modes including asexual, parasexual, and sexual reproduction, each of which have specific benefits and limitations (Zhang et al., 2021a). For *A. fumigatus* the sexual cycle was detected *in vitro* in 2009 by O’Gorman et al. (2009), who showed that *A. fumigatus* possesses a fully functional sexual cycle and described the teleomorph *Neosatorya fumigata*. The sexual cycle involves the fusion of two haploid nuclei of opposing mating-types MAT1-1 and MAT1-2 to produce a diploid zygote from which haploid spores are produced by meiotic cell division. Sexual mating with meiotic recombination increases genetic variation among progeny enabling the fungus to adapt and survive in changing environments. It also enables the fungus to get rid of deleterious mutations in contrast to strictly asexual species that instead are expected to gradually accumulate deleterious mutations, also named the Muller’s ratchet (Muller, 1964). Although the 50:50 distribution, or balancing selection, of mating types in environmental *A. fumigatus* isolates suggests that niches are present in nature that support sexual reproduction, to date such niches have not been reported and mating has only been achieved under laboratory conditions (Poggeler, 2002; Dyer et al., 2003; Varga and Toth, 2003; O’Gorman et al., 2009). Moreover, mature cleistothecia (fruiting bodies) were obtained after six months of incubation on a parafilm sealed oatmeal agar plate at 30°C in the dark, recently the supermaters were discovered, which require much shorter time to produce cleistothecia around 4 weeks (Sugui et al., 2011) and the authors were still unable to induce a sexual cycle on any other media or conditions (O’Gorman et al., 2009). It remains unclear why so specifically oatmeal agar and incubation in the dark has been successful for sexual reproduction of *A. fumigatus*, especially considering it will be difficult to identify a natural environment resembling these conditions. Most fungal species reproduce asexually when conditions are favorable for growth and reproduce sexually in nutrient-limiting conditions, which is in contrast to what has been observed for *A. fumigatus* so far (Dyer et al., 1992). The thick ascospore wall of *A. fumigatus* has been suggested to protect sexual spores from different environmental stresses including high temperature and ultraviolet radiation. Given that *A. fumigatus* is a typical soil fungus it can explain the induction of sex in the absence of light (Sugui et al., 2011).

Sexual recombination has been suggested to play a role in triazole resistance development in *A. fumigatus*, as the environmental route involves resistance mutations characterized by complex mechanisms such as a TR in combination with one or more SNPs in the *cyp*51A-gene (i.e. TR_34_/L98H (Zhang et al., 2017). As triazole resistant isolates that harbor either the TR or the *cyp*51A-SNP are extremely rare, selection of TR-mediated resistance mutations through asexual reproduction is unlikely and has only been reported once in a clinical case (Hare et al., 2019). As such complex changes may have developed through meiotic recombination, we hypothesized that sexual recombination can play a role in the development of TR-mediated resistance mutations in *A. fumigatus*. Over the past years we have investigated *A. fumigatus* population dynamics in plant-waste storage heaps, especially those produced by flower bulb growers, which may contain high levels of triazole fungicide residues (Zhang et al., 2017; Schoustra et al., 2019a). Although samples from these sites were shown to contain very high *A. fumigatus* colony forming unit (CFUs) per gram (Schoustra et al., 2019a), supporting active asexual reproduction, several observations indicated that a sexual cycle might also be present in these hotspots. A study performed by our group in 2017 revealed the presence of TR_46_-variants with three repeats $\left({\text{TR}}_{46}^{3}\right)$ instead of two copies in the promoter region of the *cyp*51A gene as a possible result of unequal crossing over during meiosis (Zhang et al., 2017). Further indications of sex in composting flower bulb waste material were not a clonal expansion but a high genetic diversity found among *A. fumigatus* isolates recovered from these heaps and the fact that significant growth of *A. fumigatus* CFUs was recorded after a 70°C heat shock, a temperature that is known to kill asexual conidia. The question now arises whether flower bulb waste stockpiles or compost heaps can provide the conditions required for sex in *A. fumigatus*. In the current study we set out to find evidence for sexual reproduction of *A. fumigatus* in flower bulb-waste and to determine if this material could provide conditions that support sexual recombination.

## Material and Methods

### Flower Bulb Waste Material and *A. fumigatus* Isolates

Decaying flower bulb waste material used in this study was obtained from flower bulb farmers in the north of the Netherlands as part of previously conducted longitudinal sampling studies (Zhang et al., 2017; Zhang et al., 2021b). All samples were previously screened for *A. fumigatus* growth as part of those studies on agar plates with and without triazole supplementation (**Tables 1**, **2**). For the sexual crossing experiments samples from six flower bulb waste stockpiles were selected that originated from different parts or stages of the composting heap that all showed triazole-resistant *A. fumigatus* growth and one sample was selected that showed no *A. fumigatus* growth. For the direct detection and visualization of ascospores in flower bulb waste material eight samples were selected that all showed significant triazole resistant *A. fumigatus* growth after a one-hour heat shock at 70°C. *A. fumigatus* isolates AfIR974 and AfIR964 from (Sugui et al., 2011) were used to set up sexual crosses.

**Table 1:** Sample characteristics of selected flower bulb waste material samples for sexual crossing experiments from (Zhang et al., 2017; Zhang et al., 2021b).

Sample	Farmer	Type of sample	Weight (grams)	*Aspergillus fumigatus* growth		
				No azole CFU/gr	TEB (4mg/l) CFU/gr	ITR (4mg/l) CFU/gr
1	A	Mature flower bulb compost	3.0-4.0	426	320	240
2	A	Flower bulb waste on agricultural field	1.5-2.0	400	300	0
3	A	Mature flower bulb compost	2.0-3.0	1,600	1,600	1,600
4	A	Top of flower bulb waste heap	2.0-3.0	44,700	4,000	4,400
5	A	1m inside flower bulb waste heap	1.5-2.0	515,000	410,000	350,000
H4	D	0.5 m inside flower bulb waste heap	2.0-3.0	560,000	515,200	43,000
45	C	Fresh flower bulb waste material	5.0-6.0	0	0	0

TEB, tebuconazole; ITR, itraconazole; CFU, colony forming units.

**Table 2:** Sample characteristics of selected waste material samples with Innosieve grid detection results of ascospore detection.

Sample	Farmer	Type of sample	*Aspergillus fumigatus* growth			Ascospore count
			No azole CFU/gr	TEB (4mg/l) CFU/gr	ITR (4mg/l) CFU/gr	
32	A	Residual flower bulb material	600,000	600,000	600,000	5
54	B	Flower bulb material	600,000	600,000	600,000	0
55	C	1m inside flower bulb waste heap	1,000,000	400,000	340,000	3
61	C	1m inside flower bulb waste heap	950,000	950,000	950,000	0
62	C	1m inside flower bulb waste heap	980,000	980,000	980,000	0
74	C	1m inside flower bulb waste heap	1,200,000	1,100,000	1,100,000	0
78	C	1m inside flower bulb waste heap	1,100,000	400,000	430,000	4
82	C	Before turning flower bulb heap, 1m inside	760,000	22,000	13,000	4

TEB, tebuconazole; ITR, itraconazole; CFU, colony forming units.

### Heat Shock Survival

It is assumed that conidia are heat sensitive and do not survive a 70°C heat shock of one hour, while ascospores can survive such a heat shock. To test this, we determined the survival of conidia (asexual) and ascospores (sexual) after a 70°C heat shock in flower bulb waste material and in saline solution. Conidial suspensions were made from an orange color mutant *A. fumigatus* isolate (Schoustra et al., 2019b) and ascospore suspensions were made from ascospores harvested from a cleistothecia from a standard crossing (oatmeal agar) of *A. fumigatus* isolates AfIR974 and AfIR964 (16 weeks old culture). Suspensions were counted and different dilutions were prepared to be added to either 1 gram of sterilized waste material in 9 ml saline solution (8% NaCl in MilliQ) or directly added to 9 ml saline solution only. Due to the different color of conidia (orange) in contrast to ascospores (green) both dilutions could be combined and because of different expected heat survival rates the following number of spores were used; 10^3^, 10^4^, 10^5^, 10^6^ or 10^7^ for conidia and 10^2^, 10^3^ or 10^4^ for ascospores. The different suspensions were tested in duplicate, heat shocked for 24 hours at 70°C in a water bath and 50 µl of the suspension was plated out at 0, 1, 2, 4, 6 and 24 hours on a Malt Extract agar plate at 37°C for 48 hours and colony forming units (CFU) were counted. Measurements of the viability of the ascospores was calculated *via* CFU count on the plate divided by hemocytometer count and the effect of orange mutant spore color on conidial survival upon heat was also tested. Due to the heat resistant nature of ascospores an even longer heat shock was additionally performed on 10^4^/ml ascospore suspensions. Suspensions were heat shocked in either of 1 gram of sterilized waste material in 9 ml saline solution (wet heat shock) or directly in sterilized waste material (dry heat shock) for up to 7 days and 50 µl was plated for counting CFU on a Malt Extract agar plate at 37°C for 48 hours on day 1, 2, 3, 4 and 7.

### Direct Detection and Visualization of Ascospores in Flower Bulb Waste Material

To demonstrate that *A. fumigatus* can undergo a sexual cycle in flower bulb waste, microscopic examination for the presence of ascospores was performed on selected samples. For this, a flow-through method for rapid capture and detection of microorganisms was used that has been developed by Innosieve Diagnostics BV^®^ (Wageningen, The Netherlands). This method relies on three steps; 1) sample preparation that ensures separation of microbes from clogging agents in the sample, 2) filtration and subsequent retainment of microbes on a dedicated membrane and staining, and 3) optical automated scanning of the membrane for the presence of specific microbes using a MuScan™ device and MuScan™ Image Analysis software for microbe location and number. This approach had been shown successful for the detection of *Salmonella enterica* in milk samples (Nguyen et al., 2015) and fungal spores in greenhouses, but required optimization for detection of *A. fumigatus* ascospores in compost. For *A. fumigatus* a specific staining was developed to distinguish conidia from ascospores, with a green dye that stained *A. fumigatus* conidia (Solophenyl Flavine 7GFE500) (Hoch et al., 2005) and a red dye (Innosieve Buffer F, IP rights by of Innosieve BV^®^) that stained *A. fumigatus* ascospores. The protocol was tested for -reactivity using fungal species that can be expected in flower bulb waste and have a similar spore size to *A. fumigatus*, including *Botrytis cinerea, Penicillium* species*, Alternaria solani* and *Fusarium graminearum* (see **Table S1**). For the MuScan™ software to correctly judge the size and shape of *A. fumigatus* spores, a microsieve grid was spiked with either conidia or ascospores. Scanning Electron Microscopy (SEM; Electron Microscopy Centre, Wageningen University and Research Centre) was used to confirm size differences and structures of *A. fumigatus* conidia and ascospores. The samples were mounted on a SEM stub by carbon adhesive glue (EMS Washington USA) and subsequently coated with 12nm Tungsten (Leica MED 020). Samples were analyzed at 2 KV, 6 pA, in a field emission SEM (Magellan 400, FEI, Eindhoven, the Netherlands). After full optimization and validation of the *A. fumigatus* grid-based detection in soil samples, eight independent flower bulb waste samples that showed *A. fumigatus* growth after heat shock from the previously published longitudinal study were analyzed (Zhang et al., 2021b).

### Sexual Crosses Using Flower Bulb Waste Material as a Nutrient Source

In this study two sexual crossing experiments were set up on flower bulb waste agar plates and in flower bulb waste tubes. For the flower bulb waste agar plates, petri dishes (15 cm) were filled about half with autoclaved waste material and 15 ml of 2% agarose water medium and left to solidify with the top surface just covered by the agar. Per waste sample three plates were made and the total weight of waste material per petri dish was recorded for each plate before adding the 2% agarose water medium (**Table 1**). For the waste material tubes, 2 grams of sterilized waste material of sample #5 was used but now directly put into sixteen glass tubes without the addition of any agarose water medium. The agar plates and glass tubes were inoculated with the so called super-mater *A. fumigatus* isolates AfIR974 and AfIR964 that can undergo a relatively fast sexual cycle by showing sexual fruiting bodies or cleistothecia after about roughly six weeks of incubation at 30°C in the dark (Sugui et al., 2011). Inoculation was done by adding 1 µl of a spore suspension of either mating types of AfIR974/964 of 1*10^5^ conidia/ml in four sectors of the agar plates as described by O’Gorman et al. (2009). In the waste material tubes, 50 µl of 1*10^7^ conidia/ml of a mixture of the isolates AfIR974 and AFIR964 were inoculated by pipetting on the inner sides of the glass tube with the intention to make future cleistothecia visible for inspection from the outside of the glass tube during incubation. Plates were sealed with parafilm and both plates as well as the glass tubes were incubated at 30°C in the dark.

## Results

### Heat Shock Survival

**Figure 1:**
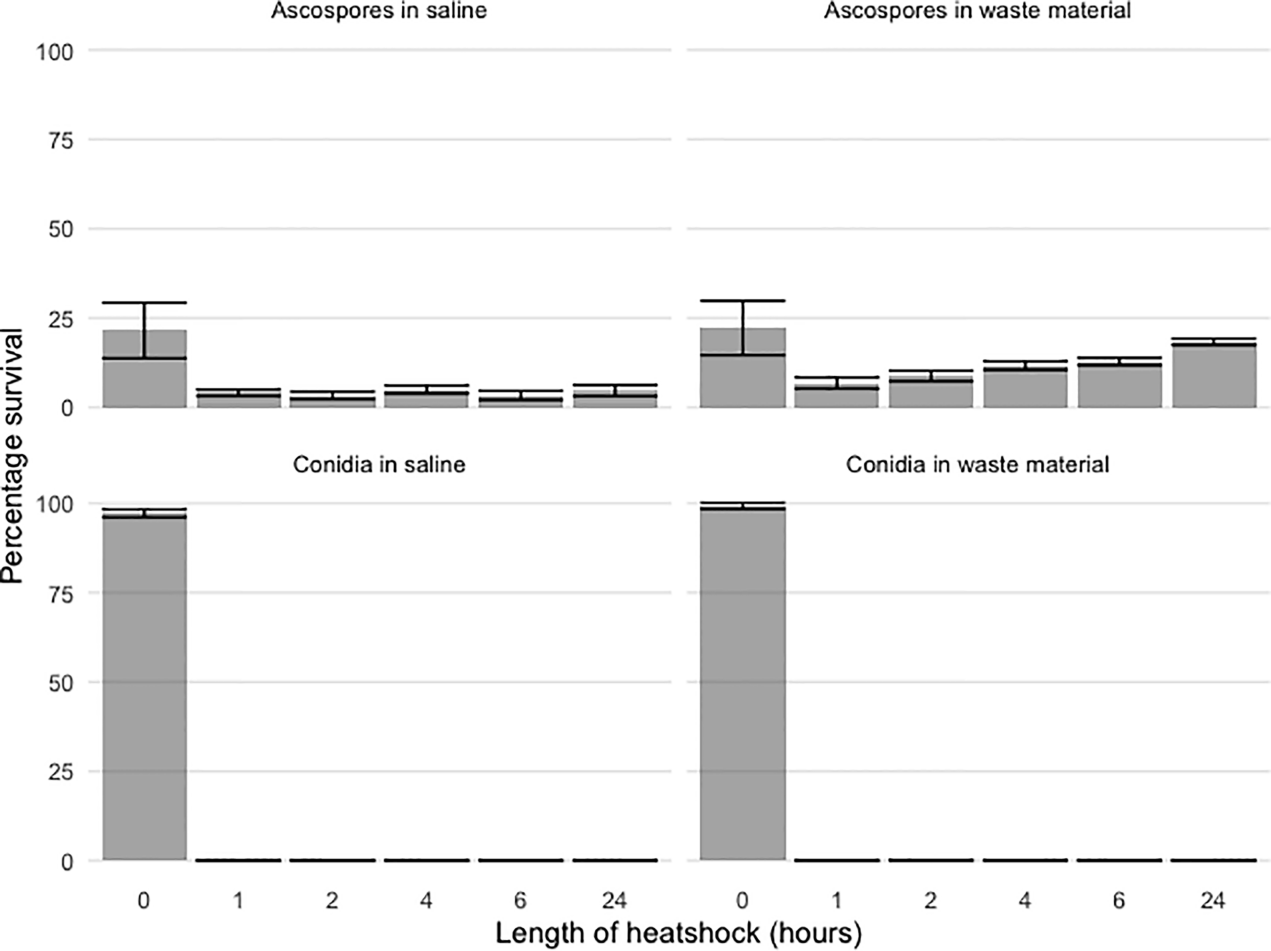
Percentage survival of spiked ascospore and conidial after 70°C heat shock of 1h, 2h, 4h, 6h, 24h. The datapoints represent colony counts of single measurements of biological duplicates and depicted is the mean +/- SEM as indicated with the error bars. Both *A. fumigatus* ascospores and conidia were spiked at different concentrations ranging from 10^3^ to 10^7^ for conidia and 10^2^ to 10^4^ for ascospores in either saline (left panels) or flower bulb waste material (right panels). Percentage survival is depicted for ascospores (upper panels) and conidia (lower panels) and show clear differences; while conidia cannot survive a one-hour heat shock, ascospores are initially decreased after the first one-hour heat shock but especially for the spiked ascospores in flower bulb waste material, germination is rising back again to the initial levels that were detected before the start of the heat shock. The initial peak of viability in the two upper panels is due to condia attached to the fruiting body wall.

**Figure 2:**
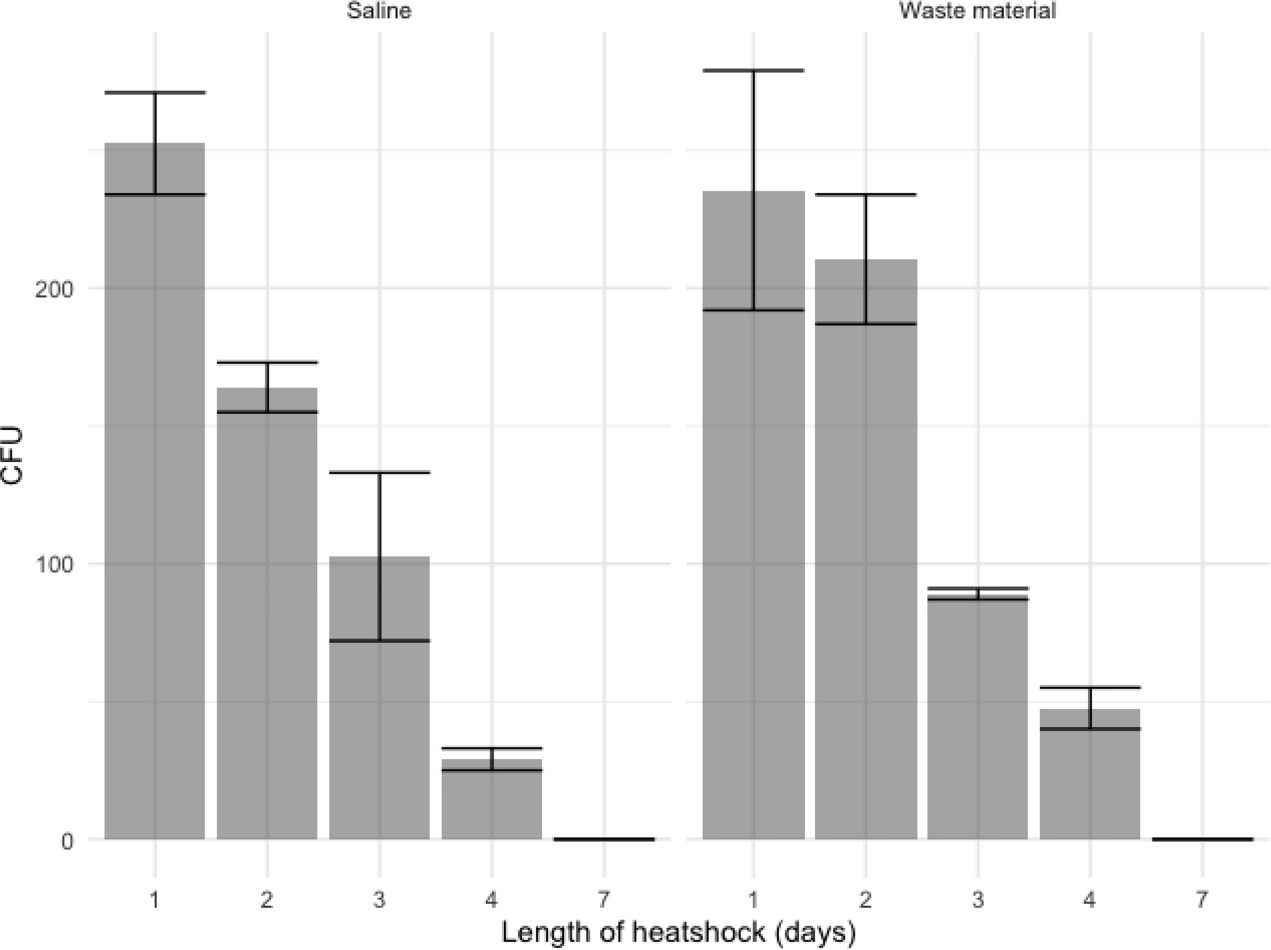
Ascospore growth after several days of heat shock at 70°C, datapoints represent colony counts of single measurements of biological duplicates and depicted is the mean +/- SEM as indicated with the error bars. With an initial inoculation of 10^4^ ascospores in saline (left) and flower bulb waste material (right), about 250 CFU are detected after 24 hours of heat shock. Ascospores show germination up to 4 days of continuous heat shock but not after seven days of heat shock.

Different concentrations of conidia and ascospores were tested for survival of a 70°C heat shock. Suspensions were made in flower bulb waste material solutions to resemble the circumstances of a stockpile. To observe if the nature of the matrix or liquid suspension influences the exposure and effect to the heat shock; saline solutions only were compared with saline solution with flower bulb waste material as well as a dry sample of flower bulb waste material only. Conidia showed an expected 100% viability by direct plating of the samples at timepoint 0. While ascospores, that are known not to exhibit a 100% viability, show for this crossing a 25% viability. For conidia, >99.9% were killed within the first hours of the heat shock (**Figure 1**). At one and two hours few viable conidia were counted from the highest concentration of spiked samples corresponding with less than 0.0004% survival. All samples taken after two hours of heat shock showed no growth and all conidia both in waste material as well as in saline were killed (**Figure 1**). An additional experiment was performed to identify whether the color of *A. fumigatus* has an effect on the survival upon heat and showed that all orange and green conidia were killed after one hour with no significant differences (data not shown). The ascospores spiked samples showed growth even after 24 hours of heat shock (**Figure 1**). The survival of ascospores but not conidia of a 24-hour heat shock at 70°C was confirmed in our experiments, yet the survival dynamics at such high temperature heat shock is not known, therefore an additional heat shock experiment was done on ascospore solutions only and now for up to seven days germination of *A. fumigatus* in flower bulb waste material solutions as well as in dry flower bulb waste material, better resembling the circumstances of a compost heap. These results showed that with an inoculum of 10^4^/ml ascospores in 10 ml of saline, flower bulb waste material solution or dry flower bulb waste material, the ascospores remained viable at least up to four days of heat shock, but not longer than seven days (**Figures 2**, **S1**).

### Direct Detection and Visualization of Ascospores in Flower Bulb Waste Material

**Figure 3:**
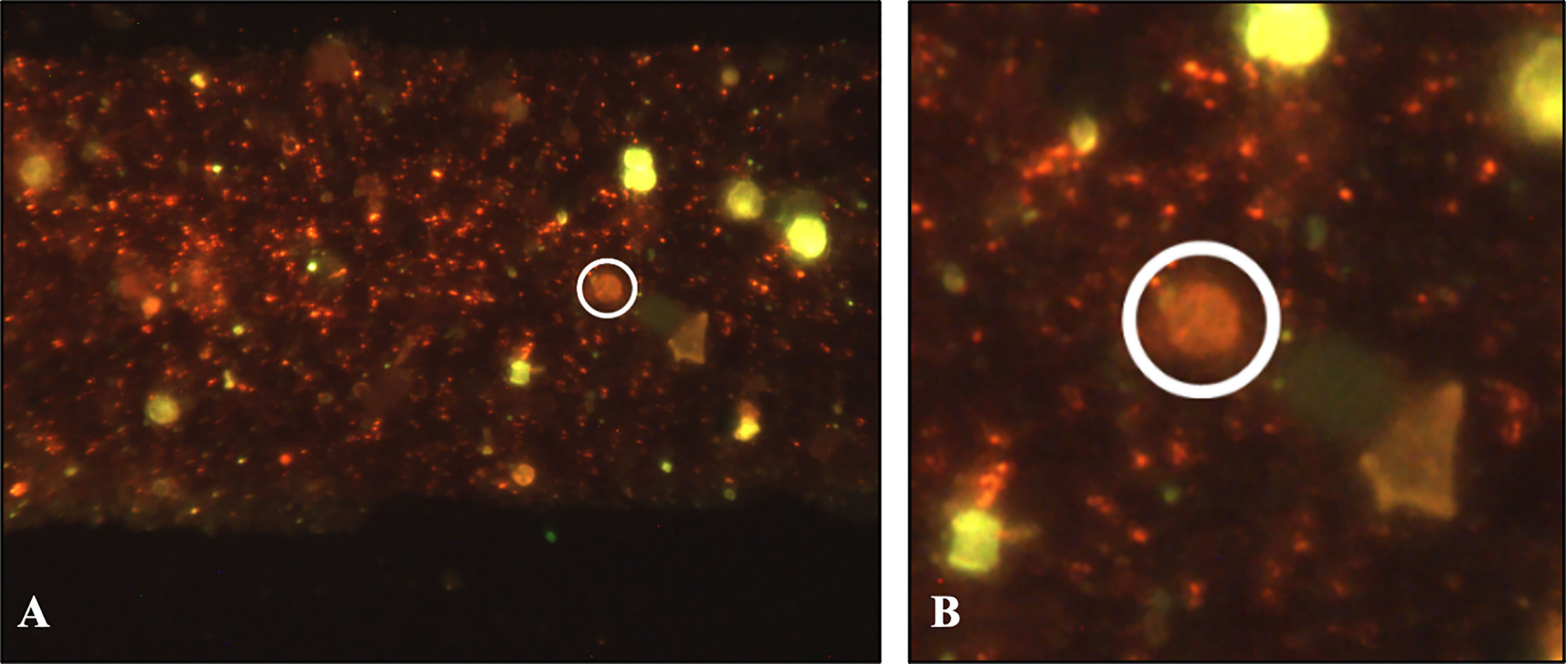
Innosieve grid detection by using an *A. fumigatus* specific fluorescent staining, the MuScan camera is able to identify both conidia (green fluorescence, **A**) as well as ascospores (red fluorescence, in white circle, **A** and enlargement **B**) within the dense layer of a flower bulb waste material (sample #78).

Using the flow-through method for capture and detection of ascospores, a microsieve grid was spiked with conidia and ascospores of *A. fumigatus* to correctly apply the MuScan software for spore detection. SEM was performed on one of the ascospore spiked grids as shown in supplemental **Figures S1A, B**, as well as on a grid with flower bulb waste material after sample preparation (**Figures S2C, D**). SEM itself is not effective for identifying ascospores in waste material because the waste material is too dense for single spore detection from the surface only (**Figure S2D**). The SEM image showed a thick layer of material that precluded single particle identification. By using a fluorescent fungal staining, however, the MuScan system is able to identify both conidia as well as ascospores specific for *A. fumigatus* within the layer of waste material (**Figures 3A, B**). Cross reactivity testing was performed using fungal species expected in flower bulb waste material and with similar spore size compared to *A. fumigatus*. Some of these species stained positive for the green dye yet could easily be distinguished from *A. fumigatus* by the MuScan software based on the size of these spores. For the red dye only the ascospores of *A. fumigatus* stained positive. All eight samples tested positive for *A. fumigatus* conidia with the specific green staining and corresponding size, four of these eight samples also tested positive for *A. fumigatus* ascospores, with three to five ascospore structures per sample (**Table 2**). The ascospores stained had the expected size, and a surface with two equatorial crests, consistent with *A. fumigatus* ascospores (**Table 2** and **Figures 3A, B**).

### Sexual Crosses Using Flower Bulb Waste Material as a Nutrient Source

**Figure 4:**
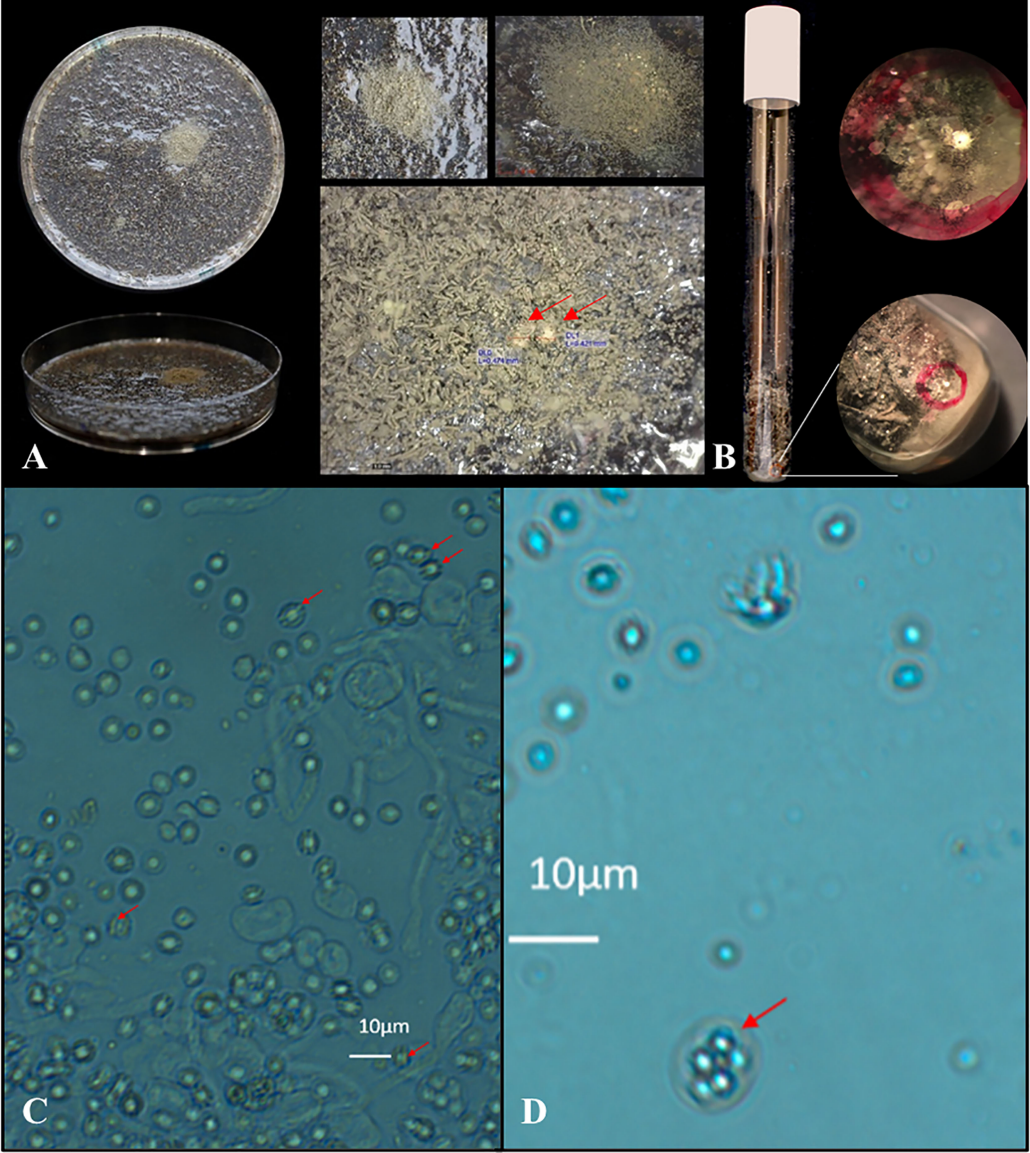
Visual and microscopic detection of cleistothecia formed on flower bulb waste material. The petri dish crossing showed limited and patch like growth within the middle of the patch two visible cleistothecia (**A**, red arrows). In the glass tube a cleistothecia was visible by visual inspection from the outside of the tube (**B**, red circle). Cleistothecia were crushed and the suspensions showed typical ascospore phenotype by microscopic inspection with two equatorial crests (**C**, red arrows) together with occasional intact asci (**D**, red arrow) confirming a successful sexual crossing of *A. fumigatus* on sample #5 flower bulb waste material.

On one of 21 plates, cleistothecia were visible as quickly as after three weeks of incubation with flower bulb waste material (number #5). Fifteen white yellowish cleistothecia were observed in total on this plate. In contrast to regular oatmeal agar plates (where cleistothecia are visible on the barrage zone of two mating types), cleistothecia produced on the waste material agar plates were only visible in limited spots and not throughout the whole barrage zone (**Figure 4A**). We then inoculated AfIR974 and AFIR964 *A. fumigatus* isolates in flower bulb waste material (sample #5) in glass tubes without water agar to more closely mimic conditions in stockpiles. After three weeks we observed the presence of cleistothecia in 50% (8/16) of the glass tubes. In total, we observed 17 cleistothecia, either *via* visual inspection or through a stereo microscope and it cannot be excluded that more cleistothecia were present but remained undetected (**Figure 4B**). For both a waste material agar plate and a waste material tube one cleistothecium was picked up and crushed by using a needle inside an Eppendorf tube. A drop of the ascospore suspension was screened under a light microscope and both cleistothecia showed typical ascospore phenotypes with two equatorial crests together with occasional intact asci (**Figures 4C, D**). After a 24 hours heat shock of the ascospore suspension at 70°C, 50 µl was plated and *A. fumigatus* colonies grew on a Malt extract agar plate after 48 hours of incubation at 37°C, thereby confirming to be viable ascospores.

## Discussion

We provide evidence for an active sexual reproduction of *A. fumigatus* in natural substrate in the environment, i.e. flower-bulb waste. Our observations are supported by laboratory experiments that confirm that *A. fumigatus* can form cleistothecia with viable ascospores in flower bulb waste material. A first indication for sexual reproduction of *A. fumigatus* in the environment was an observation made during investigation of dynamics of *A. fumigatus* triazole-resistant phenotypes in flower bulb waste heaps with and without triazole fungicide residues. After heat shock of samples obtained from both heaps *A. fumigatus* colonies were recovered, while we assumed that all *Aspergillus* conidia would not survive such heat exposure. Furthermore, microsatellite typing of these colonies showed a high genetic diversity, that is not consistent with (clonal) asexual reproduction but could point towards sexual reproduction (Zhang et al., 2017). Although several attempts were made prior to this study to identify and make cleistothecia or ascospores visible in flower bulb waste material, we were unsuccessful due to technical limitations. In this study however, we successfully used an optically flat micro engineered membrane, developed by Innosieve Diagnostics BV^®^ to detect and selectively capture *A. fumigatus* ascospores in flower bulb waste material.

Although a 70°C heat shock has been shown to be highly effective to kill *Aspergillus* conidia, we were concerned that the matrix used, i.e. degrading flower bulb waste material, may reduce killing efficacy and post-heat shock regrowth could be due to surviving conidia rather than ascospores. This is confirmed by the saline only ascospore spiked samples that showed a lower germination rate after 24 hours of heat shock compared to flower bulb waste material spiked samples. The flower bulb waste material therefore does provide a buffering effect to the heat shock on conidia. As **Figure 1** bottom graphs of conidia spiked saline or waste material shown, we confirm that a one-hour heat shock is effective to eliminate >99.9% of the conidia, but not all. This is something to consider when experimentally investigating survival of ascospores. Additional experiments showed that ascospore survival occurs even after a heat shock as long as 96 hours. It will be interesting to test whether these results are true for all *A. fumigatus* crosses or whether the genomic background of the parental isolates can affect these outcomes (Swilaiman et al., 2020). The isolates used in this study, AfIR974 and AfIR964, are natural isolates collected from the environment in Ireland and therefore could mimic a realistic, natural crossing. Another parameter to consider, that was not tested for in this study, is the maturity of the cleistothecia that is known to affect viability of ascospores (Sugui et al., 2011). Ascospore viability can increase with the age of the cleistothecia starting at 17% at four weeks up to 95% at 20 weeks.

Finally, proof of flower bulb waste stockpiles as natural niche for sexual reproduction of *A. fumigatus* is shown with the results from the Innosieve^®^ grid-based detection. Where SEM analysis cannot be performed on the dense waste material, a solid phase cytometry sample preparation combined with a fluorescent fungal staining did confirm the presence of ascospore structures in four of eight waste material samples. Even though it was technically not possible to culture and identify the observed ascospores as *A. fumigatus*, these findings together support the occurrence of sex in *A. fumigatus* in flower bulb waste. We then tested whether flower bulb waste material can provide the required nutrients and triggers to induce a sexual cycle in *A. fumigatus.* O’Gorman *et al.* showed that *A. fumigatus* is able to undergo sexual reproduction after six months of incubation on parafilm sealed oatmeal agar at 30°C in the dark, while crosses using other media including 2% MEA (Oxoid), Czapek Dox agar and Aspergillus complete medium failed. Since then, many studies replicated these results including our laboratory by specifically using *A. fumigatus* isolates AfIR974 and AfIR964 from Sugui et al. (2011). Cleistothecia can be visible within four weeks in all cases using oatmeal agar as nutrient source. In this study, we show that a different nutrient source than oatmeal can induce a sexual cycle, namely samples from agricultural flower bulb waste stockpiles. Cleistothecia were visible as quickly as three weeks after inoculation of the mating isolates, showed the typical ascospores upon crushing the fruiting bodies and also germinating after heat shock of 70˚C. In our study only one of seven samples of flower bulb waste material was successful in inducing a sexual crossing. Sexual crossing was most frequent when inoculated in a glass tube (50%), which could be due to three-dimensional access to nutrients, compared to a flat (waste material) agar plate (<5% cleistothecia formed). Additional experiments have been conducted with other flower bulb waste material samples that we collected at a later time point. Again, we were able to induce sexual crosses on some of the flower bulb waste material samples but not all, and therefore it seems that due to the heterogenic composition of flower bulb waste material samples batch-to-batch differences are present (data not shown).

So why are flower bulb waste stockpiles beneficial for *A. fumigatus* to undergo sexual reproduction? The possible explanations are that (1) flower bulb waste provides favorable conditions for sex; several key factors for sexual reproduction under laboratory conditions that have been identified seem to be present in flower bulb waste. Stockpiles of flower bulb waste represent a multi-organic resource with inside the heap a warm, dark, low oxygen/high CO_2_ environment. *A. fumigatus* isolates show a 50/50 balancing selection of the mating type genes (Paoletti et al., 2005; Swilaiman et al., 2020), which makes the chance of finding an opposing mating type in flower bulb waste stockpiles very likely. In addition, it is believed that environmental changes affect the switching between asexual and sexual reproduction. It has been argued that in many fungi, sex occurs at the end of the growing season when the conditions for somatic growth become adverse and at this moment the cost of sex is low (Chamberlain et al., 1997; Aanen and Hoekstra, 2007). Hence, a dynamic composting process with temperature gradients (20-70°C) and gas changes might provide suitable environments to undergo sex. (2) Sex could provide benefits for the survival of *A. fumigatus* in the flower bulb waste stockpiles. During the sexual cycle heat-resistant thick walled ascospores are produced that can cope with unfavorable conditions. Many planktonic organisms produce ‘resting’ stages by sexual reproduction when the environmental conditions deteriorate, such as Daphnia (Alekseev and Lampert, 2001) and in plants, seeds can survive unfavorable conditions as well (Maynard-Smith, 1978). In fungi, the outcome of sexual reproduction is most often resting spores that are used to survive in extreme conditions and to spread. For example, the dormant ascospores of *Neurospora* are activated by fire, and then start to germinate (Henney and Storck, 1964; Macfarlane, 1970). All resting spores are thickly encysted in order to survive through stressful times. In *A. fumigatus*, the sexual ascospores have a thick cell wall and the capacity to survive adverse conditions, while asexual spores are destined to germinate quickly or to act as fertilizing agents (spermatia) (Chamberlain et al., 1997; Aanen and Hoekstra, 2007; Kwon-Chung and Sugui, 2013). Related to this ecological specialization is the tendency of sexual reproduction to be induced when the environments are harsh. In response to the dynamic but harsh composting environment, *A. fumigatus* could increase its survival rate *via* sexual reproduction. Sexual reproduction also generates genetically variable genotypes *via* recombination between parental alleles. Sex can speed up adaptation and eliminate deleterious mutations much more efficiently compared with asexual growth (Bell, 1982; Camps et al., 2012; Losada et al., 2015). Therefore, highly diverse ascospores enhance the survival rate of a fungus and can maximize its success in changing environments. A changing environment can for example be a triazole containing waste heap, where triazole containing flower bulb waste is regularly added. *Via* sexual recombination, the resistant allele can be re-shuffled and may generate new variable resistance genotypes that may be better adapted to the changing triazole environment. Our previous work showed experimental crosses of two resistant TR46 isolates producing progeny harboring the triple TR46, which conferred an increased triazole resistance phenotype compared with the ancestors (Zhang et al., 2017). Even though we did not show the azole resistant haplotypes arise as a consequence of sex in this flower bulb waste, in any environment with triazole pressure and an active sexual cycle favorable new offspring genotypes can be selected.

In this study, we provide evidence that flower bulb waste is a natural niche for *A. fumigatus* to undergo a sexual cycle, which to date had only been observed under laboratory conditions. Our observation was supported by successful crosses using flower bulb waste samples as a nutrient under laboratory conditions. Recombination of *A. fumigatus* as observed in the population structure can now be explained by active sexual recombination in nature. Furthermore, our observations may provide an explanation for the development of TR-based triazole resistance mutations in the environment.

## Data Availability Statement

The original contributions presented in the study are included in the article/**Supplementary Material**. Further inquiries can be directed to the corresponding author.

## Author Contributions

PV, JZ, AD, and ES initiated the project. PV, AD, and ES designed the methodology; JZ, AR, and ES carried out experiments and formal analyses. JZ and ES prepared the manuscript. PV, JZ, AD, and ES contributed to the commenting and editing of subsequent versions of the manuscript. All authors contributed to the article and approved the submitted version.

## Funding

This publication is partly financed by the project ‘One health consequences of circularity’ with project number GROEN.2019.002 of the research program Green III which is (partly) financed by the Dutch Research Council (NWO).

## Conflict of Interest

PV reports grants from Gilead Sciences, Thermofisher, Mundipharma, Pfizer and F2G, and nonfinancial support from OLM and IMMY, outside the submitted work.

The remaining authors declare that the research was conducted in the absence of any commercial or financial relationships that could be construed as a potential conflict of interest.

The handling editor declared a past collaboration with one of the authors (PEV).

## Publisher’s Note

All claims expressed in this article are solely those of the authors and do not necessarily represent those of their affiliated organizations, or those of the publisher, the editors and the reviewers. Any product that may be evaluated in this article, or claim that may be made by its manufacturer, is not guaranteed or endorsed by the publisher.
