# Supplementary material for: Flower Bulb Waste Material is a Natural Niche for the Sexual Cycle in Aspergillus fumigatus
Source: Front Cell Infect Microbiol. 2022 Jan 21;11:785157. doi: 10.3389/fcimb.2021.785157 (PMC8823264; doi:10.3389/fcimb.2021.785157)
Supplement: Supplementary file 1 [file DataSheet_1.pdf]

Supplemental material

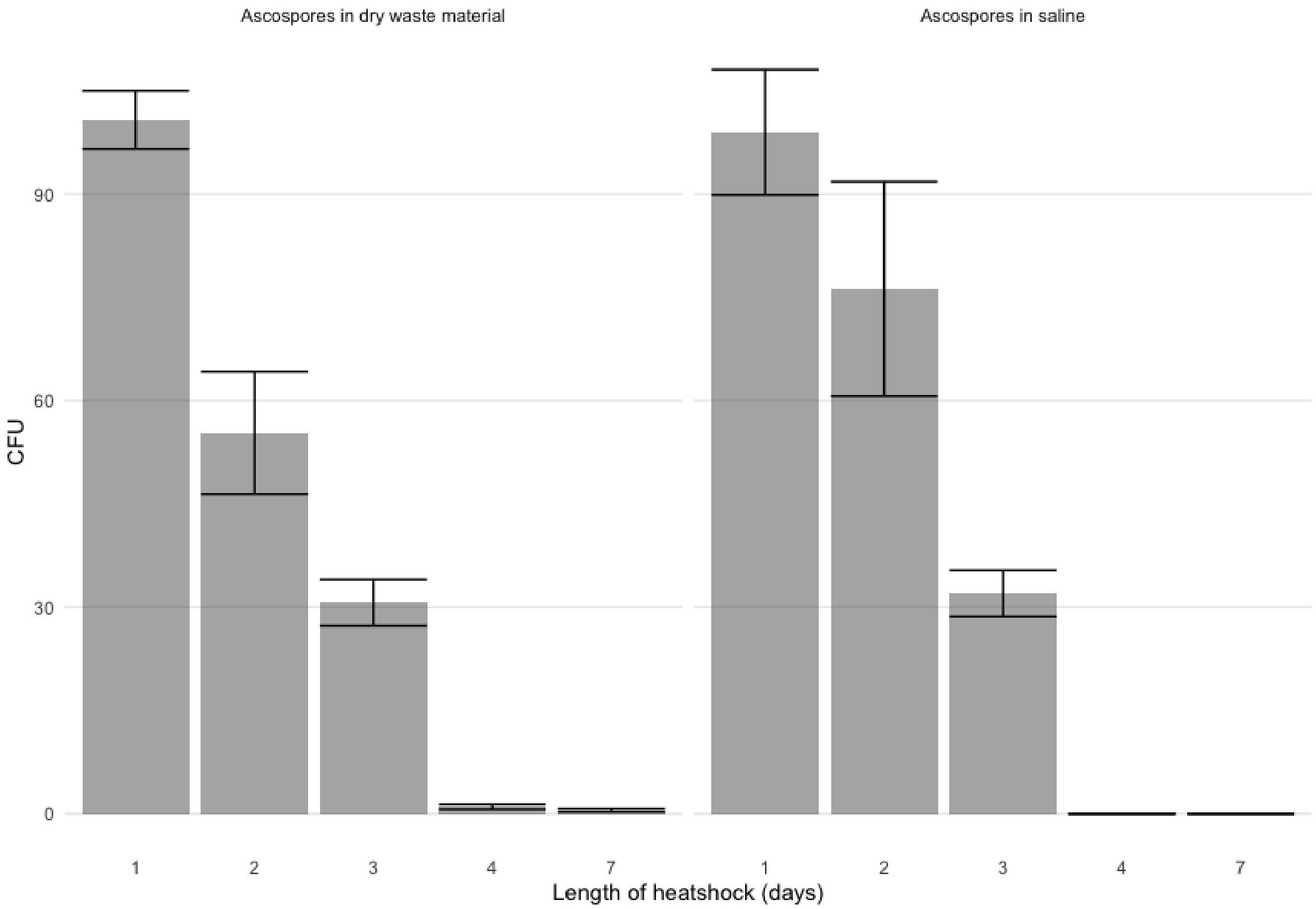

Figure S1 The heat shock experiment was done on ascospore solutions for up to seven days germination of *A. fumigatus* was detected in saline only and in dry flower bulb waste material, better resembling the circumstances of a compost heap. These results showed that with an inoculum of round $2x10^4$ ascospores into 10 ml of saline, flower bulb waste material solution or dry flower bulb waste material, the ascospores remained viable at least up to four days of heat shock, but not longer than seven days. Note: this experiment was done at different time point compared to the experiment in Figure 2

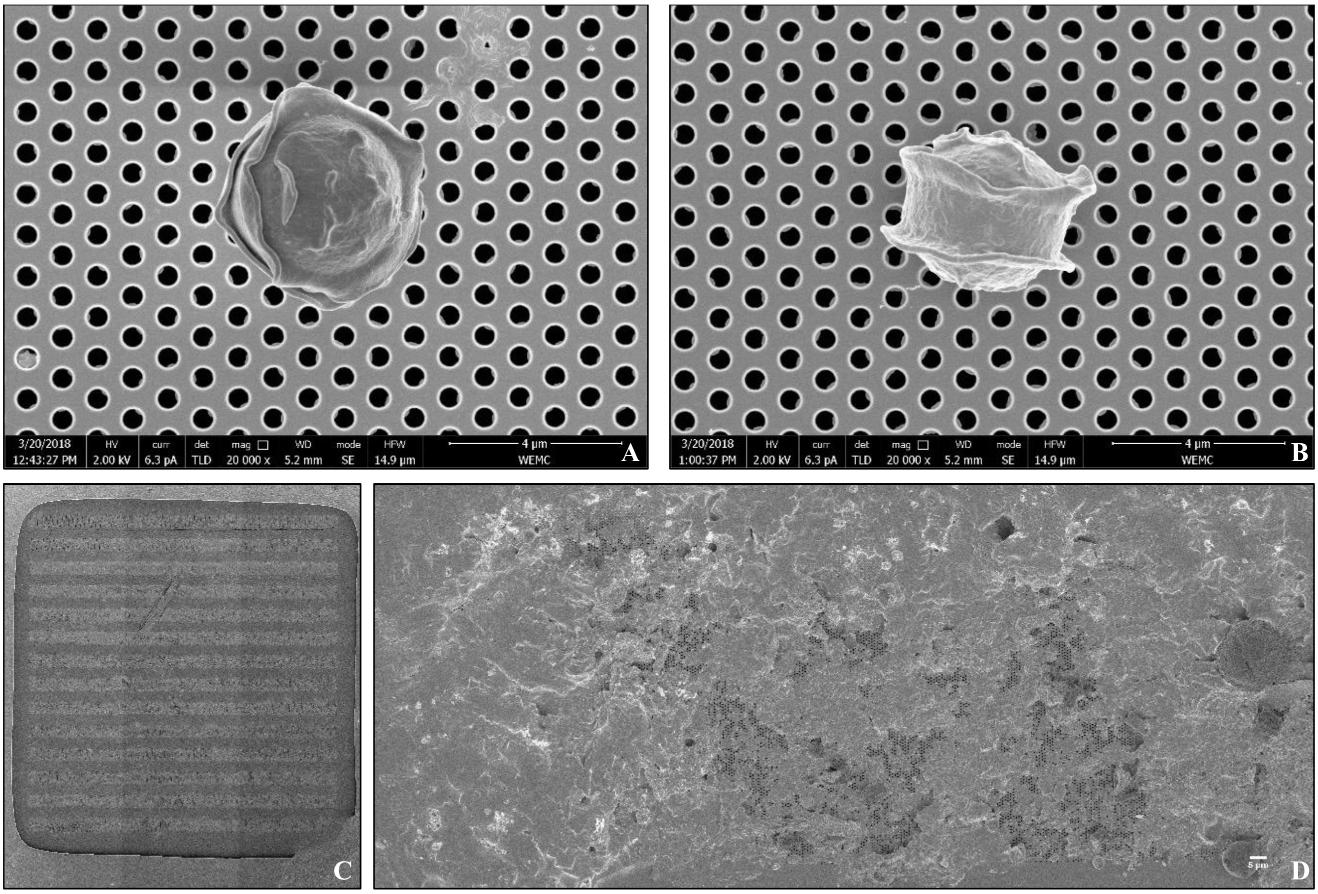

Figure S2. Innosieve grid detection. A microsieve grid was spiked with ascospores of *A. fumigatus* for calibration purposes of the MuScan software and was used for additional SEM analysis; a single ascospore is shown from the side view (A) and from the top view (B). SEM analysis was also performed on a full microsieve grid (C) and a single lane of the grid (D) containing a flower bulb waste material (sample #78) after an Innosieve sample preparation. SEM analysis itself is not effective for identifying single ascospores in flower bulb waste material simply because the flower bulb waste material is too dense (D).

Supplementary Table 1

For *A. fumigatus* a specific staining was developed to distinguish conidia from ascospores, with a green dye that stained *A. fumigatus* conidia (Solophenyl Flavine 7GFE500, Hoch et al., Mycologia, 97(3) 2005, pp580-588) and a red dye (Innosieve, Buffer F) that stained *A. fumigatus* ascospores. The protocol was tested for -reactivity using fungal species that can be expected in flower bulb waste and have a similar spore size to *A. fumigatus*, including *Botrytis cinerea, Penicillium* species*, Alternaria solani* and *Fusarium graminearum* (see Table S1)

Table S1. Staining dye specificity for *A. fumigatus* conidia, *A. fumigatus* ascospores and non-target organisms.

Organism	Applied staining dye	
	Solophenyl Flavine 7GFE500[a]	Innosieve Buffer F[b]
Saccharomyces cerevisiae yeast cell	+	+
Penicillium expansum conidia	+	N.D.
Botrytis cinerea conidia	+	N.D.
Alternaria solani conidia	+	N.D.
Fusarium graminearum macroconidia	+	N.D.
Aspergillus fumigatus conidia	+	N.D.
Aspergillus fumigatus ascospore	N.D.	+

[a]: excitation filter 470-490 emission filter: 410-550, [b]: EX 570-590 EM: 610-660. +: Detected. N.D.: Not Detected

The picture below shows the green, fluorescent conidia and the red ascospores of *Aspergillus fumigatus*. *A. fumigatus* conidia are stained with Solophenyl Flavine 7GFE500 (green fluorescent), and *Aspergillus fumigatus* ascospores are stained with Innosieve buffer F (red fluorescent).

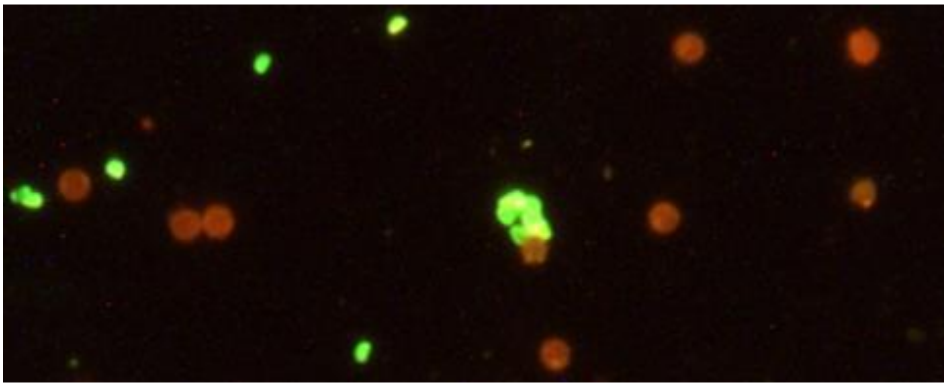

Figure S3. MuScan image from a solution containing *A. fumigatus* conidia and ascospores.
